# Clinical and Utilization Outcomes of Matched People With and Without HIV Aged 65+

**DOI:** 10.1093/geroni/igab046.3369

**Published:** 2021-12-17

**Authors:** Brianne Olivieri-Mui, Sandra Shi, Gahee Oh, Ellen McCarthy, Ira Wilson, Monty Montano, Dae Hyun Kim

**Affiliations:** 1 Hebrew SeniorLife, Boston, Massachusetts, United States; 2 Hebrew SeniorLife, Harvard Medical School, Roslindale, Massachusetts, United States; 3 Hinda and Arthur Marcus Institute for Aging Research, Roslindale, Massachusetts, United States; 4 Hebrew SeniorLife, Roslindale, Massachusetts, United States; 5 Brown University, Providence, Rhode Island, United States; 6 Brigham and Women's Hospital, Boston, Massachusetts, United States

## Abstract

The prevalence of age-standardized comorbidities is significantly elevated for PLWH across an array of cohorts. However, healthcare needs of older people living with (PLWH) and without (PWOH) HIV may be similar if they have similar geriatric conditions. PLWH and PWOH aged 65+ and eligible for Medicare from 7/1/2014-1/1/2015 were matched 1:1 on age, sex, race, and census region (n=7654). Cox regression assessed count of prevalent geriatric conditions (dementia, depression, falls, hip fracture, sensory deficits, osteoporosis, orthostatic hypotension, urinary incontinence, frailty, and polypharmacy), and risk for clinical or utilization outcomes (cancer, kidney disease, muscle wasting, hepatitis C, liver disease, myocardial infarction, stroke; hospitalization, nursing home and home health admission) during follow-up between 1/1/2015-12/31/2016. PLWH and PWOH are similar in count of geriatric conditions. Compared to those with none, those having 2+ geriatric conditions were similar across PLWH and PWOH in their risk of ≥1 clinical outcome (PLWH: HR 1.57 95% CI [1.29-1.90]; PWOH: HR 1.31 [1.02-1.67]), hospitalization (PLWH: HR 2.35 [1.96-2.83]; PWOH: HR 2.07 [1.65-2.60]), and home health admission (PLWH: HR 2.09 [1.58-2.76]; PWOH: HR 2.20 [1.55-3.12]). Having 2+ geriatric conditions, PWOH had 4.45 times the risk (95% CI 3.16-6.26) and PLWH had 2.88 times the risk (95% CI 2.18-3.81) of NH admission compared to no geriatric conditions. In this study, PLWH use nursing homes less than PWOH despite having a similar number of geriatric conditions and clinical outcomes. Further research to understand this apparent discrepancy will be critical to achieve equity in nursing home access.

